# Considering digits in a current model of numerical development

**DOI:** 10.3389/fnhum.2014.01062

**Published:** 2015-01-13

**Authors:** Stephanie Roesch, Korbinian Moeller

**Affiliations:** ^1^Leibniz-Institute for NeurobiologyMagdeburg, Germany; ^2^Knowledge Media Research CenterTuebingen, Germany; ^3^Department of Psychology, Eberhard-Karls UniversityTuebingen, Germany

**Keywords:** mathematical cognition, numerical development, embodied cognition, finger-based representation, finger counting

## Abstract

Numerical cognition has long been considered the perfect example of abstract information processing. Nevertheless, there is accumulating evidence in recent years suggesting that the representation of number magnitude may not be entirely abstract but may present a specific case of embodied cognition rooted in the sensory and bodily experiences of early finger counting and calculating. However, so far none of the existing models of numerical development considers the influence of finger-based representations. Therefore, we make first suggestions on (i) how finger-based representations may be integrated into a current model of numerical development; and (ii) how they might corroborate the acquisition of basic numerical competencies at different development levels.

## Introduction

The mental representation of number magnitude is often seen as the perfect example of what is called abstract. This seems reasonable as the quantity information conveyed by any number is independent of the characteristics of the objects in the set denoted, such as size, color, weight, etc. In line with this, Gauss wrote in a letter to Bessel in 1830 that “we must admit with humility that […] number is purely a product of our minds”—a claim which seems to be corroborated by recent data (e.g., Condry and Spelke, 2008; Cantlon et al., 2009). Nevertheless, there is also accumulating evidence in recent years suggesting that the representation of number magnitude may not be entirely abstract. Instead, it seems to not only depend on input format (see Cohen-Kadosh and Walsh, 2009 for a review and discussion) but might even represent a specific instance of embodied cognition, rooted in sensory and bodily experiences (Lakoff and Núñez, 2000; Núñez, 2004). Importantly, early finger counting has been suggested to play a vital role for the development of a representation of embodied numerosity (e.g., Domahs et al., 2010; Fischer and Brugger, 2011; Moeller et al., 2012).

On a very basic level this assumption is corroborated by the observation that the majority of children use their fingers when learning to count or calculate at some point in their numerical development (e.g., Fuson and Hall, 1983; Fuson, 1988). Additionally, it has repeatedly been found that finger-based numerical representations still influence number processing in adults. Di Luca et al. (2006), for instance, observed that there seems to be an association between specific fingers and numbers. When asked to respond to a presented number (1–10) by pressing a corresponding key, adults’ responses were faster when the key had to be pressed by a finger which was associated to the respective number in their prototypical finger counting strategy (e.g., 1 corresponding to thumb, 2 corresponding to index finger, etc.). Another line of research evaluated in how far symbolic number processing is influenced by the respective finger counting system used. In this context, Domahs et al. (2010) contrasted possible influences of the German and Chinese finger counting system. In the German finger counting system each number from 1 to 10 is not only assigned to a specific finger but also associated with a specific finger pattern following 1-to-1 correspondence (i.e., the thumb stretched out for 1, thumb and index finger for 2, thumb, index, and middle finger for 3, etc.). This means that for numbers >5 both hands are required for the respective finger pattern (i.e., one full hand plus the thumb of the other hand for 6). In contrast, in the Chinese system this 1-to-1 correspondence only applies to numbers up to 5 whereas numbers larger than 5 are indicated symbolically using one hand only. The authors found that in a symbolic magnitude comparison task, reaction times of German-speaking participants were significantly longer when at least one of the to-be-compared numbers was associated with a finger counting pattern requiring both hands (e.g., 4 vs. 6 or 6 vs. 8). Domahs et al. (2010) argue that Chinese participants did not show this increase in reaction times because their finger counting pattern only required one hand for all numbers up to 10.

Based on these and other results, Moeller et al. (2012) suggested that finger-based representations should be considered a distinct representation of number magnitude that is automatically activated whenever we encounter a number. However, so far none of the existing models of numerical development (e.g., von Aster and Shalev, 2007; Krajewski and Schneider, 2009) considers the influence of finger-based representations. Therefore, the aim of the current article is to discuss how finger-based representations might corroborate the acquisition of basic numerical competencies and to derive first suggestions on how finger-based representations may be integrated into a current model of numerical development.

## Digits in a current model of numerical development

The currently most sophisticated model of early numerical development was proposed by Krajewski and Schneider (2009). The authors assume numerical competencies to develop on three consecutive levels through an association between non-numerical abilities such as quantity discrimination, the understanding of part-whole relations, etc. and more specifically numerical skills such as counting. The following line of argument will focus primarily on the numerical skills of the model because those should be accessible and promotable by finger-based numerical representations. In the following section, we will first argue how finger-based representations will add to each of the three levels of the developmental model of Krajewski and Schneider (2009) before we will discuss possible constraints and limitations of our propositions.

### Level I: basic numerical skills

**Figure 1:**
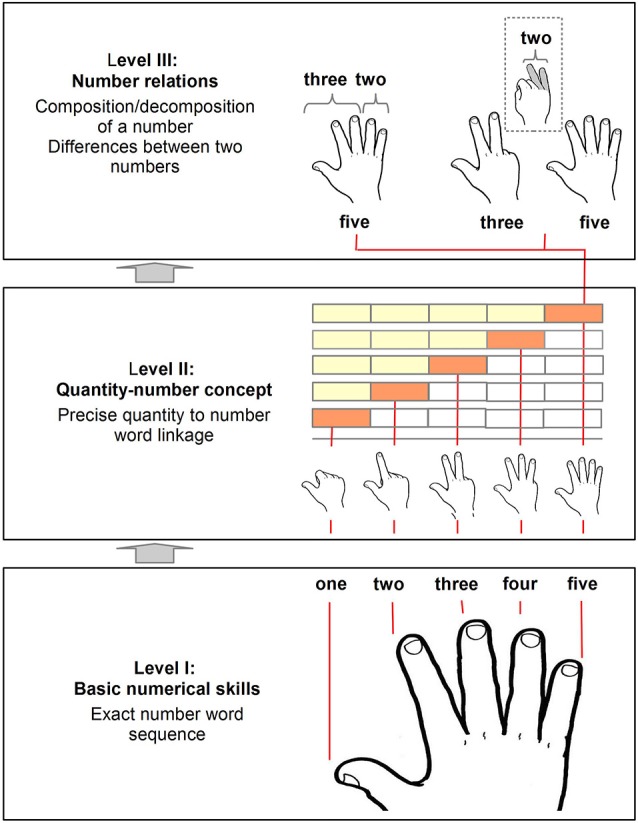
**Schematic illustration in what way finger-based representations may be integrated into the model of early mathematical development by Krajewski and Schneider (2009)**. On level I the acquisition of the number word sequence and basic counting principles can be supported by finger-based representations as each finger may be associated with a specific number word. On level II the development of quantity-number associations and of spatial-numerical representations can be corroborated because each finger pattern reflects the cardinality of the counted set. Finally, on level III the acquisition of initial calculation abilities such as number composition, decomposition, and comparison can be fostered by finger-based representations as fingers allow for grouping, regrouping, and comparing.

Krajewski and Schneider (2009) propose that on the first level of development, children learn to recite the exact number word sequence and become skilled at counting. At this stage, many of them start to use their fingers by adopting the finger counting system of the respective cultural area, even without any specific instruction to do so. In the German finger counting system, which we will refer to as the working example throughout this article, the finger counting sequence starts with the thumb for 1 and then goes on with the index finger for 2, the middle finger for 3, the ring finger for 4, the pinkie for 5, restarting the same sequence at the thumb of the other hand for 6 and so on to 10 (see Figure 1; Level I). Thus, each number word is linked to one specific finger as it is the case in most finger counting systems of Western cultures (Bender and Beller, 2011, 2012; see below for a discussion on cultural differences in finger counting). Due to this link between fingers and numbers, the counting principle of one-to-one-correspondence is easily understandable (Brissaud, 1992). On a very basic level, even the acquisition of the number words themselves might be corroborated by making use of fingers, as the finger-number association may help perceive the number words as phonological discrete items (Beller and Bender, 2011) and contributes, as some kind of marker, to memorizing them (Brissaud, 1992; Fayol and Seron, 2005; De La Cruz et al., 2014). Additionally, the counting principle of stable order and the ordinal concept of numbers might be conveyed as well (Brissaud, 1992; Fayol and Seron, 2005; Crollen et al., 2011), because the involved motor sequence during finger counting (e.g., stretching out thumb, stretching out index finger, etc.) is just as stable as the number word sequence. This might help understand that for instance “ten”, which is assigned to the ultimate finger counted, comes after “nine”, which is associated with the penultimate finger counted.

### Level II: quantity-number concept

On level II of the developmental model by Krajewski and Schneider (2009), children are suggested to become aware of the quantitative meaning conveyed by each number word. The acquisition of this so called cardinal number concept can also be supported by fingers (Brissaud, 1992). During finger counting, following the German finger-counting routine, digits are stretched out one after another whereas outstretched ones are not pulled in again (see Figure 1; Level II). This procedure allows for linking each number word to the corresponding quantity and for perceiving this quantity-number association both visually as well as through tactile and even proprioceptive sensations. By nature, finger counting is thus “cardinalized counting” (Brissaud, 1992) because quantities increase steadily (one by one) with every additional finger added during counting. This visualizes not only the respective cardinal values but also their progressive summation (see Figure 1; Level II). As this summation usually occurs in a specific spatial direction, it was suggested recently that finger counting might even modulate the spatial representation of magnitude, also known as the mental number line (Fischer, 2008). For instance, Pitt and Casasanto (2014) observed that just 15 min of training finger counting in leftward direction, this means (in palm up position) from the right thumb for 1 over the left pinkie for 6 to the left thumb for 10, extinguished and partially reversed the usual association of small numbers with left and larger numbers with right which was observed in Western participants (e.g., Dehaene et al., 1993). Taken together, these considerations indicate that fingers might not only contribute to the acquisition of ordinal counting but may also corroborate the understanding of cardinal quantity-number associations and the development of spatial-numerical associations (Fischer, 2008; Tschentscher et al., 2012).

### Level III: number relationships

On level III of their development model, Krajewski and Schneider (2009) argue that children start to learn that numbers not only convey quantities but also allow for describing relationships between quantities. This insight enables them to compose (e.g., 2 and 3 equals 5) and de-compose numbers (e.g., 5 is de-composable into 3 and 2) as well as to quantify the precise difference between two numbers (e.g., 3 is distinct from 5 by 2). Although these abilities already reflect initial calculations, they may nevertheless be corroborated by finger-based representations. Many children use their fingers when they start to calculate but not only, as often criticized, to keep track of items while counting up (e.g., Fuson, 1988) but also to visualize and combine the involved quantities as a whole (Siegler and Shrager, 1984). To combine, for example, 3 and 2 in this way, children might first stretch out thumb, index finger, and middle finger simultaneously; then add ring finger and pinkie, just to conclude finally that the result is 5, as all fingers of one hand are stretched out in the end. The other way round, finger-based strategies can also be used to visualize number decomposition. For this purpose, children might first display the initial quantity with their fingers (e.g., all fingers of one hand for 5) and then separate two subgroups of digits from each other (e.g., thumb, index, and middle finger for 3 vs. ring finger and pinkie for 2) by putting some digits closer together (see Figure 1; Level III; left side). In addition to compositions and decompositions, fingers might even exemplify differences between numbers. When children compare, for instance, the finger magnitudes 3 and 5 with each other, they can easily see them differ by 2, namely by ring finger and pinkie (see Figure 1; Level III; right side). This indicates that even number relationships, which are required for initial calculations, can be conveyed by finger-based numerical representations.

Taken together, these considerations strongly suggest that finger-based numerical representations are well-suited to corroborate the acquisition of basic numerical concepts such as counting as well as the understanding of cardinality and number relations.

### Possible constraints and limitations

While above considerations clearly argue for a specific role of finger-based representations in children’s numerical development, there also seem to be constraints and limitations to this account (e.g., Bender and Beller, 2011; Previtali et al., 2011 for discussions of this point).

A first point to consider is whether finger-based representations are a necessary step in children’s numerical development. This would be a very strong claim and hard to proof. Actually, Butterworth et al. (2011) observed that some indigene Australian cultures do not at all use their fingers in numerical contexts but are nevertheless able to perform simple calculations. On the other hand, Poeck (1964) reported the case of a girl born without forearms, who counted the fingers of her phantom hands to solve simple arithmetic problems. Additionally, Crollen et al. (2011) found that even blind children use their fingers to count and calculate, yet less often and systematically. Against this background, we are confident that finger-based numerical representations can corroborate the acquisition of basic numerical concepts, although we do not wish to claim that they are mandatory or even necessary to develop basic numerical concepts.

A second point to consider is whether above mentioned advantages of finger-based numerical representations may be generalizable across culturally differing finger counting routines. Between cultures, finger counting routines vary, for instance, (i) in the finger on which counting is started (i.e., thumb for 1 in Germany, index finger for 1 in the US, little finger for 1 in Iran); (ii) how the finger counting sequence continues from 6 to 10 (i.e., with the same finger sequence on the second hand in most Western cultures or with the first hand using finger symbolic gestures in China). In our opinion, it seems reasonable to assume that the acquisition of basic numerical concepts (i.e., counting, understanding of cardinality and number relations) may be corroborated best, when fingers and numbers are associated in 1-to-1-correspondence and a stable finger sequence is used. Thus, it should not matter which finger (on which hand) is used to begin and how the finger counting sequence continues because these two preconditions allow for both, associating (i) a specific finger with a specific number; and (ii) a specific finger pattern with a specific cardinality. In case of the Chinese finger counting system, for instance, this is only fulfilled for numbers up to 5. For numbers exceeding 5, which are represented symbolically (see above), the order of the counting sequence is still stable but there is no 1-to-1-correspondence between fingers and numbers. Therefore no finger is specifically associated with number 7, nor is the cardinality of 7 reflected in its associated finger pattern, which makes decompositions and compositions impossible (see also Domahs et al., 2012 for the case of the Korean finger counting system).

Finally, recent evidence indicates that finger counting habits vary not only across cultures but also on the individual level. Wasner et al. (2014a) observed, for instance, that whether German participants started counting on their left or right hand was influenced reliably by whether both of their hands were equally available. Additionally, Wasner et al. (2014b) found that finger patterns differed—at least for specific numbers (e.g., 4)—when participants were asked to either count to that number (i.e., by thumb, index, middle, and ring finger) or to show the respective number as a spontaneous finger pattern (i.e., index, middle, ring finger, and pinky). Irrespective of the fact that there tends to be some flexibility in adults’ finger counting habits, it seems reasonable that children benefit most, if they stick to a stable motor sequence when learning to count on fingers (for similar results on object counting see Kamawar et al., 2010). Importantly, this is corroborated by a recent study on cognitive robotics, in which De La Cruz et al. (2014) observed “that learning the number words in sequence along with [stable] finger configurations helps the fast building of the initial representation of number in the robot” and “the internal representations of the finger configurations themselves […] sustain the execution of basic arithmetic operations” (p. 1).

Nevertheless, it should be noted that all studies on the differences of finger-based representations across individuals as well as cultures were conducted primarily with adult participants. To the best of our knowledge, there is currently no study investigating the flexibility of children’s finger counting habits. Thus, further research is needed to clarify how children develop finger-counting habits and whether different finger counting routines impact on numerical development differentially.

## Conclusion

In summary, above considerations clearly suggest that finger-based representations can corroborate the acquisition of basic numerical concepts at all three levels of the developmental model proposed by Krajewski and Schneider (2009): They seem to be helpful for (I) learning the number word sequence and basic counting principles because numbers and fingers are associated in 1-to-1 correspondence during finger counting; (II) understanding the quantity-number association and developing a spatial-numerical representation because during finger counting numbers are not only associated with specific fingers but also with finger patterns that indicate the progression of magnitudes; and (III) acquiring initial calculation abilities such as composition and decomposition or number comparison because fingers allow for grouping, regrouping and comparing. Notwithstanding these reasonable benefits, we do not intend to claim that finger-based representations are a mandatory or necessary prerequisite of successful numerical development. Nevertheless, they can positively influence the acquisition of basic numerical concepts. Against this background, it seems plausible that numbers may not be “purely a product of our minds”, as suggested by Gauss in his letter to Bessel but in fact reflect a specific case of embodied cognition that roots in the bodily experiences of early finger counting and calculating. Therefore, we strongly suggest that digits should be considered in current models of numerical development.

## Conflict of interest statement

The authors declare that the research was conducted in the absence of any commercial or financial relationships that could be construed as a potential conflict of interest.
